# Widening the knowledge of non-employment as a risk factor for suicide: a Norwegian register-based population study

**DOI:** 10.1186/s12889-023-16084-x

**Published:** 2023-06-20

**Authors:** Carine Øien-Ødegaard, Lars Johan Hauge, Kim Stene-Larsen, Solveig Tobie Glestad Christiansen, Espen Bjertness, Anne Reneflot

**Affiliations:** 1 Division of Mental and Physical Health, Norwegian Institute of Public Health, Oslo, Norway; 2 Department of Community Medicine and Global Health (Department of Health and Society), University of Oslo, Oslo, Norway

**Keywords:** Suicide risk, Non-employment, Unemployment, Welfare use, Health problems, Registry data, Discrete time event history analysis

## Abstract

**Background:**

There is a known association between employment status and suicide risk. However, both reason for non-employment and the duration affects the relationship. These factors are investigated to a lesser extent. About one third of the Norwegian working age population are not currently employed. Due to the share size of this population even a small increase in suicide risk is of importance, and hence increased knowledge about this group is needed.

**Methods:**

We used discrete time event history analysis to examine the relationship between suicide risk and non-employment due to either unemployment or health-problems, and the duration of these non-employment periods. We analyze data from the Norwegian population registry from 2004 to 2014, which includes all Norwegian residents in the ages 19–58 born between 1952 and 1989. In total the data consists of 1 063 052 men and 1 024 238 women, and 2 039 suicides.

**Results:**

The suicide risk among the non-employed men and women is significantly higher than that of the employed. For the unemployed men, the suicide risk is significantly higher than the employed within the first 18 months. For the unemployed women we only find a significant association with suicide risk among those unemployed for six to twelve months. The suicide risk is especially increased among those with temporary health-related benefits. In the second year of health-related non-employment men have eightfold and women over twelvefold the OR for suicide, compared to the employed.

**Conclusion:**

There is an association between non-employment and suicide risk. Compared to the employed both unemployed men and men and women with health-related non-employment have elevated suicide risk, and the duration of non-employment may be the driving force. Considering the large share of the working age population that are not employed, non-employment status should be considered in suicide risk assessment by health care professionals and welfare providers.

**Supplementary Information:**

The online version contains supplementary material available at 10.1186/s12889-023-16084-x.

## Background

Work life can provide purpose, community, economic security, and often personal development. However, almost one out of three of the Norwegian working age population is not employed [1]. Non-employment can lead to a feeling of social alienation, especially if it is involuntarily. Lack of purpose and low level of social integration, as well as financial difficulties, are all associated with non-employment, and are also known risk factors for suicide [2]. About 650 lives are lost to suicide in Norway each year, and worldwide suicide is one of the leading causes of loss years of life [3, 4].

Being unemployed is associated with an increased risk of suicide [5, 6]. However, unemployment only refers to those in search for a job. In addition, there are multiple groups who also lack employment, such as those that are permanently non-employed, stay home parents, and those that are not employed due to health-related causes etc. The suicide risk among several of these sub-groups is scarcely investigated. Considering the number of people experiencing non-employment, the impact of increased suicide risk in these groups may be considerable, and investigating suicide risk in these under-examined sub-groups is vital [1, 7]. Some studies have examined the relationship between unemployment and suicide, and they are consistent in establishing a relationship [5, 6, 8–11]. However, whether this is due to job loss/unemployment itself or varies with duration of unemployment is unclear. There are studies that argue for the impact of duration [8, 12–14], but some of them are outdated. For instance, all the studies included in the meta-analysis by Milner et al. (2014) have unemployment records from the 90’s [14]. The suicide risk among people who experience non-employment due to health problems is far less investigated. Most of the studies concerned with health-related non-employment have investigated disability pensioners, which is usually a permanent status [15–18]. This article focuses on people on temporary benefits, and thus recipients of permanent welfare benefits are excluded.

Herein, we aim to expand the understanding of the association between non-employment and suicide risk by focusing on two groups receiving welfare instead of labor income: the unemployed and those with temporary welfare benefits due to health problems. Studies from different societies find that non-employment can increase the suicide risk [6, 8, 9, 15, 18], but the severity of the consequences of not being employed is likely to be impacted by the society of which it occurs. It is of interest to investigate whether such an association persists even in a country like Norway which has a strong welfare state, with economic assistance that probably lessens the economic strain of being without labor income. In 2017 about one third of the working age population received welfare for subsistence instead of income in Norway [19]. We have included all temporary health-related welfare benefits in the period 2004 to 2014. To our knowledge, this is the first study to investigate the suicide risk among recipients of these types of benefits. The first objective of the present study is to estimate the suicide risk associated with the duration of unemployment. The second objective is to investigate the suicide risk among those on temporary welfare benefits due to health problems. By doing this, we are expanding the knowledge regarding suicide risk among two groups of the non-employed. Investigating group differences is a prerequisite for targeted suicide prevention measures.

## Material and methods

### Study design

We analyze data from several Norwegian administrative registers linked with unique personal identification numbers. We have linked data from the Cause of Death Registry, the Norwegian Population Register, Statistics Norway’s Educational Registration System, FD-trygd (Social security database) from the Norwegian Labour and Welfare Administration (NAV) and constructed individual record linkages. All the registers are updated annually, except for the data from the Social security database, which is updated with every change in social security. To match the registers, we have subtracted one record per person per year.

We use discrete time event history analysis to investigate the risk of suicide mortality. As we have annual data, models utilizing discrete time events are appropriate. The method exploits the timing of events, not only their occurrence. It is also appropriate to assess the impact of events, like exogenous covariates and duration. It is also particularly suited for efficiently exploiting the wealth of information embedded in merged administrative register data. The results in this type of analysis are given in odds ratios (ORs). However, when dealing with rare events, like suicide, the results are quite similar to risk ratios. In addition to estimating the ORs, we calculated the predicted probabilities for suicide and plotted these. The benefit of estimating these marginal effects is the possibility of comparison across models, whereas the results from the discrete time event history analysis only allow for within-model comparison.

The maximum observation time is eleven years, with individuals censored out in the case of other causes of death or emigration. All Norwegian residents in the ages 19–58 born between 1952 and 1989 are included. In total the data consists of 1 063 052 men and 1 024 238 women, and the total of 20 518 005 person-years. The observation period is from 2004 to 2014. We used Stata 16.0 to conduct the analyses.

### Variables

The outcome variable is death by suicide in any given year. Suicide victims are identified by the ICD-8 and ICD-9 codes E950–E958 and the ICD-10 codes X60–X84, Y870. The variable is coded 0 for all the years prior to the year of which the suicide occurs, and then shifts to 1 in the year of the suicide. The informant is censored the following year. The variable has the value 0 in all records for the non-suicide victims.

There are two different exposure variables, according to type of non-employment. The first of these is being unemployed. This variable represents everyone who receives unemployment benefit. To be eligible for this welfare, either loss of income or loss of at least 40% of working hours must be experienced, as well as having earned a minimum salary the previous months [20]. Depending on previous earnings it is possible to have unemployment benefit up to two years. This variable is categorical and grouped into the following six: “Employed”, “0–3 months”, “3–6 months”, “6–12 months”, “12–18 months”, and “18–24 months”, depending on the duration of the unemployment period. Each person has only one record per year, as stated above, indicating the length of unemployment that year. It is a time-varying variable, with the “Employed” as the reference category.

The second exposure variable is health-related non-employment. This variable denotes those who are currently outside the labor market due to health problems and receive temporary health-related welfare benefits.

In Norway people on sick leave can remain in employment during the first year of absence and cannot be fired due to the medical condition during this period. Since we focus of the non-employed this group is excluded from the analyses. Our topic is the health-related welfare benefits received by those with an illness that has lasted for more than one year, and have therefore exhausted their right to sick pay, but who do not (yet) qualify for disability pension. In the period 2004–2010 this included a number of different welfare benefits (time-limited disability pension, rehabilitation allowance and rehabilitation benefit). In March 2010 these benefits were merged and replaced by a single benefit called work assessment allowance (AAP) [21]. To qualify for these welfare benefits the ability to work must be reduced by 50%. The recipients of these types of welfare often receive several consecutive types of health-related benefits. This means that although each benefit is time-limited to a few years, some beneficiaries receive health-related benefits for much longer [22]. Some of the recipients never return to the labor market, but rather end up with disability pension [22]. The variable for the duration of the health-related benefit is a categorical time-varying variable, divided into four groups: “Employed”, “One year”, “Two years” and “Three to four years”. The time intervals are longer in this model than for the unemployed because the health-related benefits are granted for longer periods of time. The reference category is the employed.

The comparison group in both models are men and women aged 19–58 born between 1952 and 1989, that are employed. To qualify as employed one must work a minimum of one hour per week. Everyone who is employed and receive health-related benefits at the same time are categorized with the controls. This may decrease the results, but part time employment probably provides several of the positive effects of employment, like social integration, feeling of purpose and financial benefits. In each of the analysis of the two groups of non-employed, the other type is excluded.

Educational level is a confounder and is thus included in the analyses as a threefold time-varying categorical variable. The levels are “Elementary school”, “High school” and “Higher education”. To be categorized into one of them, that level of education must be completed. The reference category is “High school”.

Age is categorized into five-year age groups, “19–23”, “24–28”, “29–33”, “34–38”, “39–43”, “44–48”, “49–53” and “54–58”. This is also a time-varying variable. The reference category is “34–38”.

There is a well-documented link between family situation and suicide risk [23–25], and there is probably also a relationship between family situation and employment status. Marital status is thus included as a control variable. Marital status is a time-varying factor variable and coded as “Never married”, “Married”, “Widowed/widower”, “Divorced” and “Separated”, where “Married” is the reference category.

A categorical variable with a group for each year the individual occur in the dataset is included to control for variations in follow up-time.

In the results section below, we will present the ORs of the exposure variables, but not the other covariates.

## Results

### Distribution of the dataset

Table 1 displays the distribution of the dataset on the exposure variables, as well as the covariates educational level and marital status. The first column shows the distribution of the suicide victims, while the second column presents the distribution of the rest of the dataset. The victims of suicide are overrepresented among both the unemployed and those with health-related non-employment. However, the discrepancy is far greater in the health-related non-employment group than the unemployed. Those who die by suicide are also underrepresented in the highest educational level group and overrepresented in the lowest, compared to the non-suicide victims. The difference is small among those with high school as their highest completed educational level. There are also much fewer of those who die by suicide who are married. Although the dataset is very close to evenly divided by sex, almost three quarters of the suicides are male. Therefore, we conducted analyses separately from men and women.

**Table 1:** Distribution of the dataset

*Exposure*	Proportion and number of suicide deaths	Proportion of controls
Unemployed	5.7% (*N* = 116)	3.4%
Health-related non-employment	21.4% (*N* = 425)	3.0%
Employed	73.7% (*N* = 1 503)	96.0%
*Educational level*		
Elementary school	33.8% (*N* = 689)	18.3%
High school	43.9% (*N* = 896)	43.7%
Higher education	22.3% (*N* = 454)	38.0%
*Marital status*		
Never married	57.5% (*N* = 1 173)	46.1%
Married	22.8% (*N* = 465)	43.2%
Widow/widower	0.5% (*N* = 10)	0.5%
Divorced	13.3% (*N* = 271)	8.2%
Separated	5.9% (*N* = 120)	2.1%
*Sex*		
Men	73.2% (*N* = 1 493)	51.0% (*N* = 1 063 052)
Women	26.8% (*N* = 546)	49.0% (*N* = 1 024 238)
Number of suicides/observations	100% (*N* = 2 039)	100% (*N* = 20 518 005)

*Legend:* Percentage of the suicide deaths within each category at the time of death. Percentage of the total person-years observations within each category, except for the suicide victims

### Unemployment

In men, the ORs for suicide among the unemployed is significantly increased for all time-periods up to 18 months, as compared with employed (Table 2). There is a non-significant increasing trend of higher suicide risk with time. After one year of unemployment, the OR is above twice as high as that of the employed. Only one of the estimates regarding women is statistically significant, the unemployed for six to twelve months. This estimate, however, shows over double the OR for suicide among the unemployed women, compared to the employed.

**Table 2:** Odds ratio for suicide among unemployed men and women actively searching for employment, compared to the employed

Unemployed	Men, *N* = 10 157 286		Women, *N* = 9 692 711	
	OR	95% CI	OR	95% CI
0–3 months	1.79**	1.20 – 2.67	1.86	0.77 – 4.49
3–6 months	1.88**	1.24 – 2.84	1.14	0.37 – 3.56
6–12 months	1.94**	1.30 – 2.89	2.51*	1.24 – 5.08
12–18 months	2.38**	1.43 – 3.96	0.56	0.08 – 4.01
18–24 months	0.76	0.36 – 1.61	1.61	0.60 – 4.32

*Legend:* Controlled for education level, marital status, age group and number of records. All results are relative to the employed men and women

^*^p < .05; **p < .01; ***p < .001

**Fig. 1:**
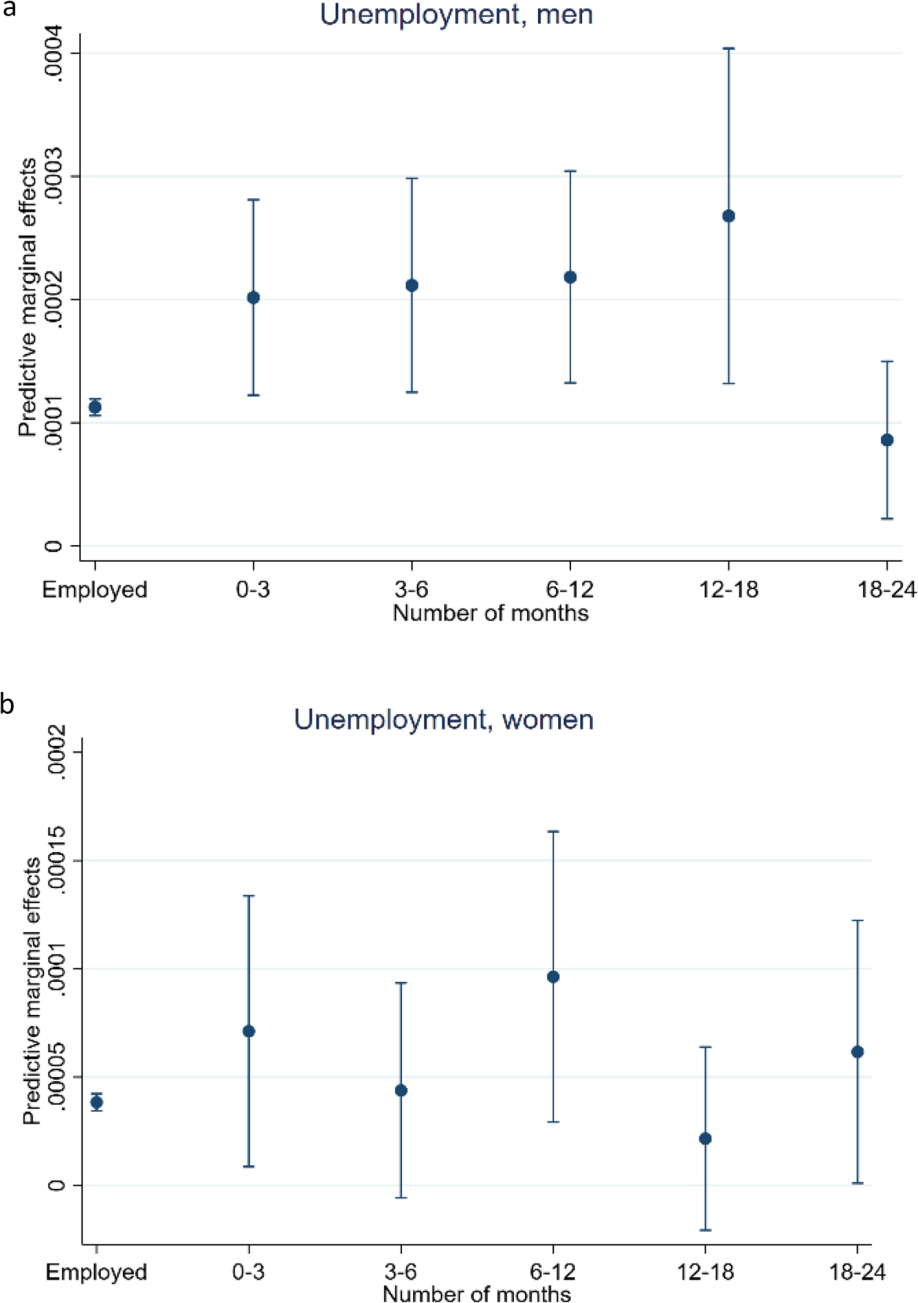
**a** Unemployment, men. Predictive marginal effects on suicide among unemployed men, compared to the employed. **b** Unemployment, women. Predictive marginal effects on suicide among unemployed women, compared to the employed

Figures 1a and b are made from the models that resulted in the estimates presented in Table 2. The figures show the marginal change in predicted probability for death by suicide across the months of being unemployed, compared to the employed. While the results in Table 2 are relative, these are absolute, and thus comparable. Figure 1a shows that in the first 12 months there is an absolute increase in predicted probability for suicide among unemployed men, compared to the control group, but it is rather small. After the first year, however, the increase is not significantly different from the predicted probability for the controls, but it is significantly higher than 0. After 18 months there can be a decline back to the same level as the population controls, but this estimate is not statistically significant from 0. In Fig. 1b, the predicted probability for suicide within six to 12 months is seemingly not significantly different from the employed. However, test of parameter estimation shows evidence at the 5% significance level that the predictive margins differ for employed and women unemployed for six to 12 months.

### Health-related non-employment

The ORs for death by suicide among men and women with health-related non-employment is displayed in Table 3. Compared to men and women in the control group, the OR for suicide is very high. The first year, men have almost six times as high OR as the ones employed. It might be an increase with time. All the confidence intervals for the estimates for men without employment are overlapping and are thus not significantly different from each other.

**Table 3:** Odds ratio for suicide among men and women with health-related non-employment, compared to the employed

Health-related	Men, *N* = 10 043 146		Women, *N* = 9 720 119	
	OR	95% CI	OR	95% CI
1^st^ year	5.54***	4.61 – 6.63	8.49***	6.54 – 11.04
2^nd^ year	8.02***	6.34 – 10.15	12.14***	8.70 – 16.95
3-4^th^ year	8.16***	6.13 – 10.87	13.70***	9.37 – 20.03

*Legend*: Controlled for education level, marital status, age group and number of records. All results are relative to the employed men and women

^*^p < .05; **p < .01; ***p < .001

Women with health-related non-employment have almost ninefold the OR for suicide as female controls. After two successive years the OR is higher, over 12 times as high as for women in the control group. As with men with health-related non-employment, there might be an increase with time.

**Fig. 2:**
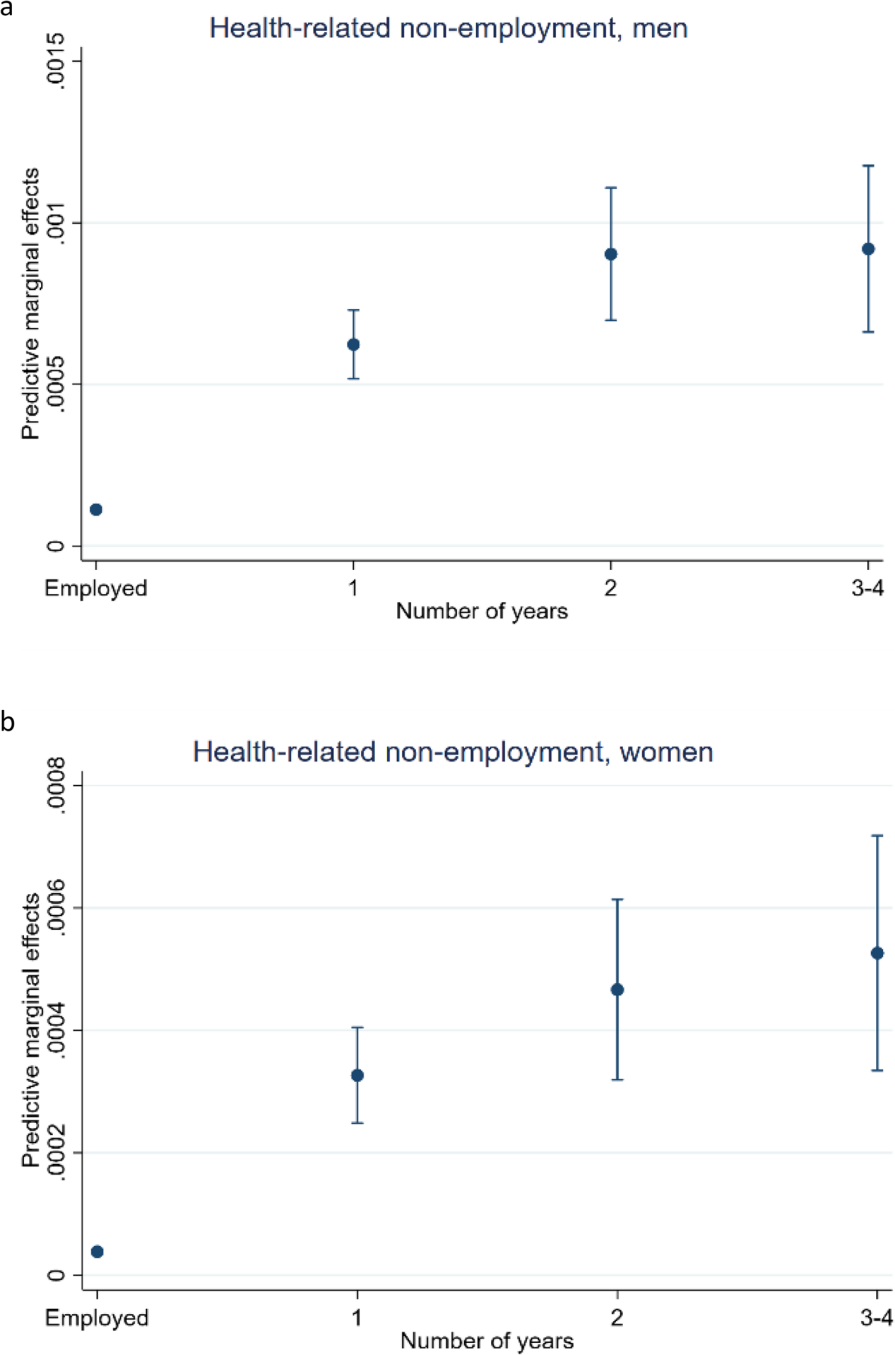
**a** Health-related non-employment, men. Predictive marginal effects on suicide for men with health-related non-employment, compared the employed. **b** Health-related non-employment, women. Predictive marginal effects on suicide for women with health-related non-employment, compared the employed

Figures 2a and b show that there is a substantial increase in predicted probability for suicide after one year of health-related non-employment. Both men and women have even higher predictive probability after two or more years, although the confidence intervals overlap. For men the predicted probability after two years is almost 0.001 (almost 1 out of 1000), and for women it is 0.0005. Although the predicted probability for suicide in general is low, this is a *considerable* rise in suicide risk, both in absolute terms, compared to the employed, and compared to the unemployed in Figs. 1a and b. Women with health-related non-employment have an even higher increase in suicide risk than unemployed men. Figure 2a show that the estimate is about as high after three to four years for men. For women it might even be an increase, as presented in Fig. 2b.

## Discussion

Overall, our results indicate that being unemployed or experiencing health-related non-employment is associated with higher suicide risk, compared to the employed. Men and women with health-related non-employment have a considerable elevated suicide risk the first year, and the non-significant increasing trend indicates that duration is the driving force in the increment.

A systematic review that investigated the association between long-term unemployment and risk of suicide found that the highest risk is within the first five years [12]. The estimation of predicted probabilities enables comparisons across models. Men with health-related non-employment have much higher predicted probabilities for suicide than unemployed men, but the results show a lasting elevated suicide risk. Unemployed women do not have significantly higher predicted probability for suicide than employed women, but women with health-related non-employment have even higher predicted suicide risk than unemployed men. As women contribute to a substantial share of those experiencing non-employment due to health problems, this underlines the importance of the results presented in this study.

We found a significant association between unemployment and suicide risk among women who were unemployed for six to twelve months, but not for the other time intervals. This result can have several explanations. In general, women have a lower employment rate than men [26]. As it is more common for women it may be less stigmatizing than for men. It might also be that women can have an “alternative career” in having and raising children, while providing for the family is a stronger ideal among men. We did conduct analyses with “any child” included as a dummy variable for both sexes, but it did not add anything new to the results.

Classen and Dunn [13] argue that unemployment duration is the dominant force in the relationship between job loss and suicide risk. Their results suggests that those who have been unemployed for less than five weeks do not have statistically significant different suicide risk than the employed [13]. This applies to both sexes. However, those who have been unemployed for 15–26 weeks have a higher risk of suicide [13]. Following this, their results suggest that it is not the job loss itself that may increase the suicide risk. Whether this is due to financial difficulties, loss of purpose, network or status, loss of confidence in new employment, or other factors are unknown. Their results, however, are obtained in a society with very different support system than the Norwegian. Our results indicate elevated suicide risk already in the first time-interval for men, and as our first interval is longer than five weeks, we cannot exclude that the job loss itself also contributes to the increase. However, we also find higher estimates among those with longer unemployment periods. For women it is only those unemployed for at least six months who have a significant higher risk of suicide. This may also mean that for women it is not the job loss, but duration that drives this positive association. Correspondingly the suicide risk seems to increase with time among those with health-related non-employment. It may be that duration of non-employment is a part of the explanation for this, but it is likely that the duration of living with a health problem is at least part of the cause. Although we cannot conclude, our results in sum lend support to their argument.

Longer duration also means larger gap since last work experience, and more time for both financial problems and mental strain to grow. The unemployed in our data receive unemployment benefits and must have been employed prior to the current non-employment period. Having this experience recently may contribute to making unemployment more short term than health-related non-employment, but the main difference between these groups is the presence of health problems.

While everyone who experience non-employment can lack a sense of meaning, cohesiveness, and personal bonds, those with health-related non-employment have the additional strain of either a mental or physical health problem. Both factors are related to increased risk of suicide, and it may be the consequence of these that is visible in the results. A Swedish study investigating the transition to disability pension (DP) in young people found that in the time leading up to the transition, the risk of attempted suicide was high [27]. In the time following the transition, however, the suicide risk declined. This can indicate that the insecurity of unstable welfare benefits and/or the strain of seeking DP may have contributed to a rise in suicide risk. Those with health-related non-employment in our dataset all have temporary benefits. This may add to the estimated suicide risk in our results.

Several previous studies have suggested a link between mental health and unemployment [14, 28–30]. Two meta-analyses have examined the relationship between unemployment and suicide/mortality and found no significant difference between those studies controlling for health status at baseline and those who did not [14, 31]. Although the results do not reject the possibility that mental health can act as a confounder in the unemployment and suicide relationship, they also find support for mental health problems acting as an intermediary between employment status and suicide. In such an event controlling for mental health condition would lead to biased estimations, while not controlling for confounding variables would do the same. Following this argument, it is a close relationship between mental health status and employment status, but not as clear how to keep the bias to a minimum.

### Strengths and limitations

This paper has several strengths. The findings are based on data with very high coverage of the Norwegian working age population. All resident in Norway, for at least six months, within the right age span or birth cohort is included. Because the dataset is comprised of administrative registers, selection bias due to drop out or stigma related to mental health problems, non-employment or other problems are minimal. This kind of high-quality data is especially valuable when examining marginal phenomena, like suicide.

There are also some limitations to the results. Those without known educational status and/or marital status have been omitted from the analyses. Although immigrants and descendants of immigrants are included in the analyses, they are probably overrepresented in the omitted group. Lack of information regarding diagnoses and/or somatic or mental health problems, previous suicide attempts and other stressful life events than marital disruption also limits the results. The data are also somewhat dated, and societal factors may have changed since the observation period. However, there has not been a shift in the unemployment rate from the period under study to today [1]. The share of the working stock population who are employed has also been steady at around 70% during the observation period and until today [1]. The share of the working age population who received temporary health-related benefits was, however, a bit higher during the observation period than today [32].

Another limitation to the results is survival bias. Our results suggest a clear link between the various forms of non-employment and death by suicide, and that duration impacts these relationships. However, the duration estimates presented in this study are influenced by survival bias. As the time span of the non-employment period broadens, some of those most at risk for death by suicide will die by suicide. This will in turn make the study population on average less inclined to die by suicide. This is a clear limitation, but it is difficult to sidestep this type of survival bias in a study focusing on duration and suicide risk.

### Future research

We propose that future research build on our results with the following improvements. First, to include immigration as control variable. There are studies that point to variations in suicide risk between immigrants and native-born. Second, previous, or current mental health problems could be included in the analyses.

In addition to the unemployed and health-related non-employment there is at least one other group that is outside the labor market. These are those not engaged in education, employment, or training (NEET). This group is estimated to be 18% of the 20–24-year-olds in 2014 [33]. As suicide is the leading cause of death in young people, we propose that future research investigates the relationship between suicide risk and being NEET.

We also suggest that future studies utilize the same or similar data sources with contra factual models, enabling the researcher to come nearer a causal relationship, given that such a relationship exists. The analyses used in this paper only give correlating results. We cannot affirm any causal effects.

## Conclusion

Our results indicate a clear association between non-employment and suicide risk for both sexes. There are, however, differences according to both types of non-employment and the duration of these. Compared to the employed both unemployed men, and men and women with health-related non-employment have a lasting elevated risk of suicide.

The overall findings illustrate that a part of the population is experiencing high suicide risk over time. Considering both the emotional costs for the suicide bereaved and the societal costs of lost years of life, it is important to implement measures for reducing non-employment and thus the suicide risk. Measures like skills learning and improving work-related self-perceptions should be investigated, preferably in combination with addressing mental health issues. In addition, measures for including people with health problems should be introduced to a larger extent. Accommodated positions, buildings or work tasks may welcome more people into the labor force. For young people in particular, measures for preventing drop out in high school are also very important. Distributing information about the associations between non-employment and suicide risk, as well as implementing prevention measures, are vital. It is particularly important that clinicians are aware of this when meeting unemployed or people with health-related non-employment. As some studies suggest that unemployment insurance/unemployment benefits can reduce the negative mental health consequences of unemployment [34, 35], the implications of the results presented here may be even more serious in societies with a less developed welfare state.

## Supplementary Information

**Additional file 1.**

## Data Availability

The datasets generated and/or analyzed during the current study are not publicly available due to data protection reasons but are available from the corresponding author upon reasonable request, and with permission of the Cause of Death Registry, the Social Security Database and Statistics Norway. The data were used under license for the current study, and restrictions apply to the availability of these data.

## Abbreviations

NAV: Norwegian Labour and Welfare Administration
DP: Disability Pension
NEET: Not engaged in Education, Employment or Training
WHO: World Health Organisation
