# Supplementary material for: Widening the knowledge of non-employment as a risk factor for suicide: a Norwegian register-based population study
Source: BMC Public Health. 2023 Jun 20;23:1181. doi: 10.1186/s12889-023-16084-x (PMC10280913; doi:10.1186/s12889-023-16084-x)
Supplement: Supplementary file 1 — Additional file 1. [file 12889_2023_16084_MOESM1_ESM.docx]

| **Unemployed** | Men, N=10 157 286 | | Women, N=9 692 711 | |
| --- | --- | --- | --- | --- |
|  | OR | 95% CI | OR | 95% CI |
| 0-3 months | 1.79** | 1.20 – 2.67 | 1.86 | 0.77 – 4.49 |
| 3-6 months | 1.88** | 1.24 – 2.84 | 1.14 | 0.37 – 3.56 |
| 6-12 months | 1.94** | 1.30 – 2.89 | 2.51* | 1.24 – 5.08 |
| 12-18 months | 2.38** | 1.43 – 3.96 | 0.56 | 0.08 – 4.01 |
| 18-24 months | 0.76 | 0.36 – 1.61 | 1.61 | 0.60 – 4.32 |
| **Age**  **(ref=44 – 48)** |  |  |  |  |
| 19 – 23 | 1.01 | 0.78 – 1.30 | 0.68 | 0.41 – 1.12 |
| 24 – 28 | 0.89 | 0.70 – 1.21 | 0.67 | 0.43 – 1.04 |
| 29 – 33 | 0.82 | 0.65 – 1.03 | 1.04 | 0.71 – 1.53 |
| 34 – 38 | 0.96 | 0.77 – 1.19 | 0.77 | 0.52 – 1.15 |
| 39 – 43 | 1.05 | 0.85 – 1.29 | 0.91 | 0.62 – 1.32 |
| 49 – 53 | 0.98 | 0.80 – 1.25 | 1.13 | 0.78 – 1.63 |
| 54 – 58 | 1.11 | 0.86 – 1.44 | 1.65* | 1.10 – 2.46 |
| **Educational attainment (ref= high school)** |  |  |  |  |
| Elementary school | 1.65*** | 1.45 – 1.88 | 1.34* | 1.03 – 1.75 |
| Higher education | 0.67*** | 1.58 – 0.78 | 0.84 | 0.66 – 1.06 |
| **Marital status**  **(ref = married)** |  |  |  |  |
| Never married | 1.81*** | 1.55 – 2.12 | 2.17*** | 1.66 – 2.85 |
| Widow/Widower | 2.59* | 1.07 – 6.29 | 1.37 | 0.43 – 4.34 |
| Divorced | 2.18*** | 1.76 – 2.70 | 2.72*** | 2.00 – 3.71 |
| Separated | 4.46*** | 3.42 – 5.82 | 4.92*** | 3.18 – 7.62 |
| **_cons** | 0.00*** | 0.00 – 0.00 | 0.00*** | 0.00 – 0.00 |

Table A1: Odds ratio for suicide among unemployed men and women actively searching for employment, compared to the employed.

*p < .05; **p < .01; ***p < .001
Legend: Controlled for number of records. All results are relative to the employed men and women.

Table A2: Odds ratio for suicide among men and women with health-related non-employment, compared to the employed.

| **Health-related** | Men, N=10 043 146 | | Women, N=9 720 119 | |
| --- | --- | --- | --- | --- |
|  | OR | 95% CI | OR | 95% CI |
| 1^st^ year | 5.54*** | 4.61 – 6.63 | 8.49*** | 6.54 – 11.04 |
| 2^nd^ year | 8.02*** | 6.34 – 10.15 | 12.14*** | 8.70 – 16.95 |
| 3-4^th^ year | 8.16*** | 6.13 – 10.87 | 13.70*** | 9.37 – 20.03 |
| **Age**  **(ref=44 – 48)** |  |  |  |  |
| 19 – 23 | 1.03 | 0.80 – 1.32 | 0.60* | 0.38 – 0.95 |
| 24 – 28 | 0.93 | 0.74 – 1.15 | 0.62* | 0.42 – 0.90 |
| 29 – 33 | 0.85 | 0.69 – 1.06 | 0.89 | 0.64 – 1.24 |
| 34 – 38 | 1.06 | 0.87 – 1.30 | 0.84 | 0.61 – 1.16 |
| 39 – 43 | 1.03 | 0.85 – 1.26 | 0.85 | 0.62 – 1.17 |
| 49 – 53 | 1.04 | 0.85 – 1.28 | 1.05 | 0.76 – 1.44 |
| 54 – 58 | 1.07 | 0.84 – 1.36 | 1.42* | 1.00 – 2.02 |
| **Educational attainment (ref= high school)** |  |  |  |  |
| Elementary school | 1.44*** | 1.28 – 1.63 | 1.21 | 0.97 – 1.51 |
| Higher education | 0.68*** | 0.59 – 0.79 | 0.93 | 0.76 – 1.15 |
| **Marital status**  **(ref = married)** |  |  |  |  |
| Never married | 1.97*** | 1.69 – 2.27 | 2.46*** | 1.94 – 3.11 |
| Widow/Widower | 2.26 | 0.93 – 5.48 | 1.34 | 0.49 – 3.64 |
| Divorced | 2.46*** | 2.03 – 2.99 | 2.79*** | 2.13 – 3.66 |
| Separated | 4.23*** | 3.29 – 5.45 | 4.30*** | 2.93 – 6.33 |
| **_cons** | 0.00*** | 0.00 – 0.00 | 0.00 | 0.00 – 0.00 |

*p < .05; **p < .01; ***p < .001
Legend: Controlled for number of records. All results are relative to the employed men and women.
